# Immersion Vaccination by a Biomimetic-Mucoadhesive Nanovaccine Induces Humoral Immune Response of Red Tilapia (*Oreochromis* sp.) against *Flavobacterium columnare* Challenge

**DOI:** 10.3390/vaccines9111253

**Published:** 2021-10-29

**Authors:** Sirikorn Kitiyodom, Teerapong Yata, Kim D. Thompson, Janina Costa, Preetham Elumalai, Takayuki Katagiri, Sasithon Temisak, Katawut Namdee, Channarong Rodkhum, Nopadon Pirarat

**Affiliations:** 1 Wildlife Exotic Aquatic Animal Pathology-Research Unit, Department of Pathology, Faculty of Veterinary Science, Chulalongkorn University, Bangkok 10330, Thailand; taregust@hotmail.com; 2 Biochemistry Unit, Department of Physiology, Faculty of Veterinary Science, Chulalongkorn University, Bangkok 10330, Thailand; Teerapong.Y@chula.ac.th; 3 Moredun Research Institute, Pentlands Science Park, Penicuik EH26 0PZ, UK; janina.costa@moredun.ac.uk; 4 School of Ocean Science and Technology, Kerala University of Fisheries and Ocean Studies, Kochi 682506, Kerala, India; preetham@kufos.ac.in; 5 Laboratory of Fish Health Management, Course of Aquatic Biosciences, Tokyo University of Marine Science and Technology, Tokyo 108-8477, Japan; takakata@kaiyodai.ac.jp; 6 Bio Analysis Group, Chemical Metrology and Biometry Department, National Institute of Metrology (NIMT), Pathum Thani 12120, Thailand; sasithont@nimt.or.th; 7 National Nanotechnology Center (NANOTEC), National Science and Technology Development Agency (NSTDA), Pathum Thani 12120, Thailand; katawut@nanotec.or.th; 8 Department of Microbiology, Faculty of Veterinary Science, Chulalongkorn University, Bangkok 10330, Thailand; Channarong.R@chula.ac.th

**Keywords:** red tilapia, systemic immune response, columnaris disease, nano-immersion vaccine

## Abstract

Immersion vaccination with a biomimetic mucoadhesive nanovaccine has been shown to induce a strong mucosal immune response against columnaris disease, a serious bacterial disease in farmed red tilapia caused by *Flavobacterium columnare*. However, the induction of a systemic immune response by the vaccine is yet to be investigated. Here, we examine if a specific humoral immune response is stimulated in tilapia by a biomimetic-mucoadhesive nanovaccine against *Flavobacterium columnare* using an indirect-enzyme-linked immunosorbent assay (ELISA), serum bactericidal activity (SBA) and the expression of immune-related genes within the head-kidney and spleen, together with assessing the relative percent survival of vaccinated fish after experimentally infecting them with *F. columnare*. The anti-IgM antibody titer of fish at 14 and 21 days post-vaccination was significantly higher in chitosan complex nanoemulsion (CS-NE) vaccinated fish compared to fish vaccinated with the formalin-killed vaccine or control fish, supporting the serum bactericidal activity results at these time points. The cumulative mortality of the unvaccinated control fish was 87% after challenging fish with the pathogen, while the cumulative mortality of the CS-NE vaccinated group was 24%, which was significantly lower than the formalin-killed vaccinated and control fish. There was a significant upregulation of *IgM*, *IgT*, *TNF α*, and *IL1-β* genes in the spleen and kidney of vaccinated fish. Significant upregulation of *IgM* and *IgT* genes was observed in the spleen of CS-NE vaccinated fish. The study confirmed the charged-chitosan-based mucoadhesive nanovaccine to be an effective platform for immersion vaccination of tilapia, with fish generating a humoral systemic immune response against columnaris disease in vaccinated fish.

## 1. Introduction

*Flavobacterium columnare*, a gram-negative, filamentous, thin rod bacterium, with or without yellow rhizoid colony formation, is a serious pathogenic bacterium causing columnaris disease in intensively farmed tilapias worldwide [1,2]. *Flavobacterium columnare* infections can lead to skin lesions, fin decay, and gill tissue damage, contributing to significant economic losses and a high mortality rate [3,4]. The virulence of *F. columnare* is demonstrated by its ability to adhere to mucosal surfaces, gliding motility, biofilm formation, and capsule production, which have been associated with its rhizoid morphotype [5]. The colonization of the bacterium to the mucosal surfaces of the fish (skin and gills) is an important step in initiating the infection, disease severity, and progression, and the typical pathological characteristics associated with columnaris disease. Vaccination against columnaris disease has been trialed in a variety of fish species. However, only low or partial protection has been reported for columnaris vaccines administered by injection or immersion using formalin-killed whole cell preparations in coho salmon *Oncorhynchus kisutch* [6], channel catfish *Ictalurus punctatus* [7,8], eels [9], carp *Cyprinus carpio* [10], and tilapia *Oreochromis niloticus* [11,12]. Among the vaccination delivery routes used to administer vaccines to fish, immersion vaccination is considered to be the most suitable for delivering columnaris vaccines to the mucosal tissues to confer a protective mucosal immune response to protect fish against the disease. Nevertheless, this approach has been impeded by the fact that the effectiveness of antigen absorption by mucosal tissues is limited and the potency of induction of protective immune responses can be low and short in duration. Our previous study demonstrated the use of a biomimetic-mucoadhesive nanovaccine that allows better adsorption of antigens to the mucosal surfaces of fish [13,14]. Strong mucosal immunity was triggered by the vaccine, inducing an immune cascade at the mucosal site and in the mucosal associated lymphoid tissue (MALT) following immersion immunization [4]. However, the ability of this vaccine to activate a systemic humoral immune response has not yet been elucidated. The aim of the present study was to investigate the specific humoral immune response stimulated in tilapia by the biomimetic-mucoadhesive nanovaccine against *F. columnare,* using an indirect-enzyme linked immunosorbent assay (ELISA) to measure serum antibody responses, serum bactericidal activity (SBA), and the expression of immune-related genes within the head-kidney and spleen. The in-house ELISA developed in the study seems suitable for monitoring the specific humoral response in tilapia against the columnaris disease.

## 2. Materials and Methods

The use of animals in experimentation for this study was officially approved by the Institutional Biosafety Committee and the Institutional Animal Care and Use Committee of Faculty of Veterinary Science, Chulalongkorn University (IBC1831052; IACUC1831020). All procedures were carried out in accordance with university guidelines and regulations as well as policies governing biosafety procedures. 

### 2.1. Fish and Experimental Conditions

Six hundred red tilapia (*Oreochromis* sp.) with an average weight of 100 g, were acclimatized for 10 days and randomly placed in four 200-L fiberglass tanks (150 fish per tank) for the four treatments described below. The tanks were maintained under continuous aeration at 25–28 °C, 5.8–6.8 ppm dissolved oxygen (DO), pH 7.5–8 and less than 0.1 mg/L of total ammonia throughout the experiment. Experimental fish were fed twice a day and water was changed up to 50% every second day.

### 2.2. Bacteria and Vaccine Preparation

**Figure 1:**
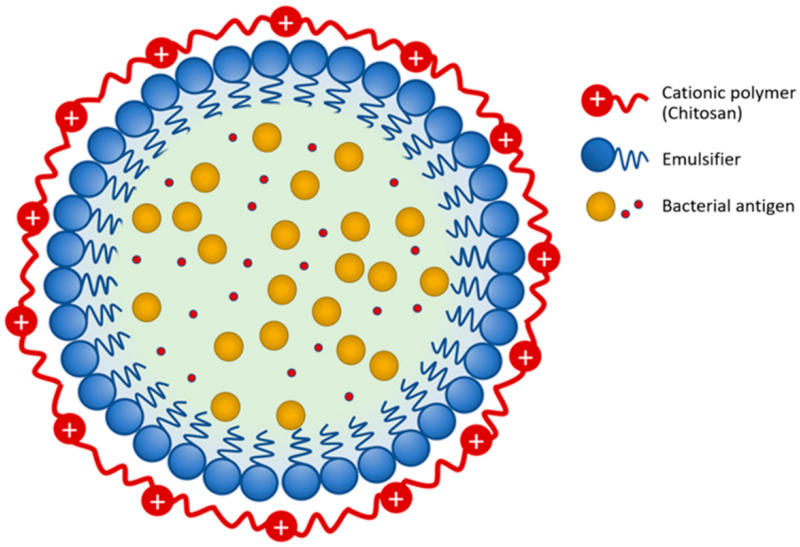
Schematic diagram of the in chitosan complex nanoemulsion vaccine.

**Figure 2:**
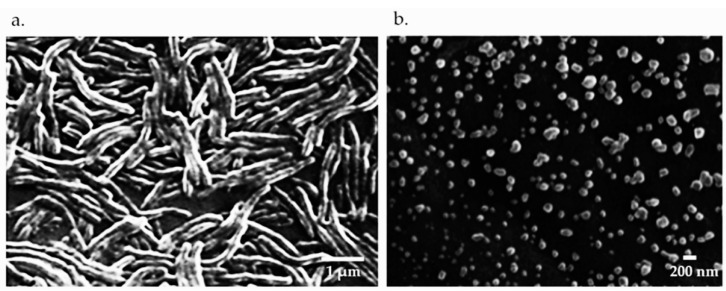
(**a**) Scanning electron microscopy (SEM) of the surface morphology of formalin killed vaccines, Scale bar = 1 μm. and (**b**) SEM images of CS-NE vaccine, scale bar = 200 nm.

*Flavobacterium columnare* isolate (F-K17/1, GenBank accession no. MW362353), used in our previous studies, was selected based on its ability to form rhizoid colonies, its high virulence in clinical outbreaks and belonging to genetic group 4 determined by 16 s rRNA phylogenetic analysis. Bacterial cultures used in the vaccine preparation were grown in Tryptone Yeast Extract Salts Agar (TYES) broth at 25–28 °C for 48 h. Bacteria were killed with 0.2% formalin and incubated at 4 °C for 20 h. Bacterial cells were collected by centrifuging at 3000× *g* at 4 °C for 30 min. Formalin-killed bacteria were washed three times with phosphate-buffered saline (PBS, pH 7.2) and the bacterial concentration of the vaccine preparation was adjusted to 10^8^ CFU mL^−1^. Formulation of the vaccine was carried out according to Kitiyodom et al. (2019) [13]. In brief, to prepare the whole cell killed bacterial vaccine (WC), an aliquot of bacterial cells (15% *w*/*w*) was mixed with PBS (85% *w*/*w*). To prepare the chitosan complex nanoemulsion (CS-NE), an aliquot of bacterial cells (10^10^ CFU mL^−1^) was sonicated at 40% amplitude for 10 min (30% *w*/*w*) was mixed with 6% (*w*/*w*) of polyoxyethylene (20) sorbitan monolaurate, 2% (*w*/*w*) of medium chain triglycerides (MCTs) and 62% (*w*/*w*) of water. The mixture was homogenized using an ultrasonic homogenizer at 40% amplitude for 5 min. The complexation of the nanoemulsion with chitosan was performed by adding 1% of chitosan (previously dissolved in 1% acetic acid) to the nano-emulsion at a ratio of 1:1 (*v*/*v*). The mixture was stirred for 1 h at room temperature. The CS-NE schematic diagram and image of scanning electron microscope (SEM) of the resulting nanoparticles are shown in Figure 1 and Figure 2. The final bacterial concentration in WC and CS-NE vaccine was 10^8^ CFU mL^−1^.

### 2.3. Vaccination and Vaccine Efficacy Test

Red tilapia (100 g) were divided into four groups: (1) whole cell killed bacteria vaccine (WC); (2) nanovaccine (CS-NE); (3) polymer blank (polymer), and (4) PBS (control) (150 fish per group, 1 tank group^−1^). Fish were immersed in the vaccine solutions, diluted 1:100 with tank water (10^6^ CFU mL^−1^) for 30 min with aeration. After vaccination, fish were transferred into fiberglass tanks containing 200 L of water. At 1, 3, 14, and 21 days after vaccination, blood was collected from 6 fish per group by caudal puncture using a 25 G × 16 mm needle and 1 mL syringe, and allowed to clot 1 h at 25 °C. The serum was collected following centrifugation at 3000× *g* for 10 min and stored at −20 °C until analyzed [11]. After 30 days post-vaccination (dpv), fish (30 from each group, 3 replicate tanks) were challenged with a lethal concentration 80 (LC_80_) of a virulent strain of F-K17/1 by immersion for 1 h. The cumulative mortality, and survival rates were recorded for 14 days after challenge and the relative percent survival (RPS) was calculated, RPS = 1 − (mortality rate of vaccinated fish/mortality rate of control fish) × 100 [15]. 

### 2.4. Serum Bactericidal Activity (SBA)

The serum from fish was prepared as described above. *Flavobacterium columnare* colonies were centrifuged and the pellet was washed and suspended in PBS. The optical density of the bacterial suspension was adjusted to an optical density of 0.8 at 540 nm (1 × 10^6^ CFU/mL). A volume of 2 µL of bacterial suspension was mixed with 20 µL of fish serum in each group and incubated at room temperature for 1 h. Phosphate buffer saline was used in place of the serum for the negative control. After the incubation, the number of viable bacteria were determined as colony forming units (CFU)/mL by plating the bacterial suspension onto TYES for 48 h at 28 °C. The bactericidal rate was calculated as follows: (1 − the number of viable bacteria after serum treatment/the number of viable bacteria after PBS treatment) × 100% [16].

### 2.5. Enzyme-Linked Immunosorbent Assay (ELISA) 

Antigen preparation: *Flavobacterium columnare* strain FK17/1 was cultured in TYES broth for 48 h and harvested by centrifuge at 10,000× *g* 4 °C for 20 min. The bacterial cells were washed three times with PBS and then resuspended in PBS. The bacterial solution was sonicated on ice at an amplitude of 45 Hz for 10 min. The total protein content of the supernatant was measured using a Nanodrop 1000 spectrophotometer (Thermo Scientific, Waltham, MA, USA), with bovine serum albumin (BSA) used as a standard [11,17]. Stock antigen preparations were stored at −20 °C until used. 

ELISA procedure: The ELISA 96-well microplates (Costar, Elmira, NY, USA) were coated with 100 µL of *F. columnare* antigen in coating antigen (0.05 M sodium carbonate buffer pH 9.6) overnight at 4 °C. Unbound antigen was discarded, and the plates were washed three times with washing buffer (PBS containing 0.05% Tween 20 pH 7.3). Non-specific bindings were blocked by adding 250 µL of blocking solution (1% *w*/*v* BSA in PBS pH 7.2) at 22 °C for 2 h. After incubation, the plates were washed three times with washing buffer. A two-fold serial dilution of serum samples was prepared in PBS and 100 µL of diluted sera was added to each well. After an overnight incubation at 4 °C, the plates were washed five times with washing buffer and were incubated for 5 min on last wash. A mouse anti-tilapia (*O. niloticus*) IgM monoclonal antibody (Aquatic Diagnostics Ltd., Oban, UK) was used to quantify the specific antibody response in fish sera, diluted in PBS according to the manufactures protocol, using 100 µL well^−1^ and incubating at 22 °C for 1 h. The plates were washed five times with washing buffer, incubating for 5 min on the last wash. After which, 100 µL of anti-mouse IgG- Horseradish peroxidase HRP (KPL, Gaithersburg, MD, USA) was used as the secondary antibody at a 1:2000 dilution, and the plate was then incubated at 22 °C for 1 h. The plates were washed five times with washing buffer and were incubated for 5 min on the last wash. Tetramethylbenzidine chromogen was added to each well (100 µL^−1^) and incubated for 5 min at 22 °C. The reaction was then stopped by adding 50 µL stop solution to each well. The absorbance of the plates was read using a microplate reader at 450 nm. Results are reported as optical density (OD) at 450 nm. All samples and controls were run in duplicate.

Standardization of the indirect ELISA method: The indirect ELISA conditions were standardized by chessboard titration using serial dilutions of sonicated *F. columnare* cells tested against serial dilutions of positive and negative tilapia sera [17]. The antigen was coated onto the ELISA plate using bacterial concentrations of 1.25, 2.5, 5.0, 10.0, and 20.0 μg/mL. Tilapia sera were diluted with PBS at dilutions of 1:20, 1:40, 1:80, 1:160, 1:320, 1:640, and 1:1280. The mouse anti-tilapia IgM monoclonal antibody was also diluted with PBS at dilutions of 1:4000. The chessboard titration method was conducted using sera from tilapia challenged with *F. columnare* as positive samples and non-challenged tilapia as negative samples. The optical density values derived from the method were then calculated (Supplementary Table S1). 

Calculation of cut-off value: The cut-off value was obtained by measuring the optical density (OD) at a wavelength of 450 nm. The cut-off OD values were calculated from the mean of the negative control (15 fish, 2 replicates) plus 3 standard deviations, as described previously [17]. Screened fish serum with an OD value greater than the cut-off value were considered as seropositive. Fish sera with an OD value lower than cut-off value were considered as seronegative.

**Figure 3:**
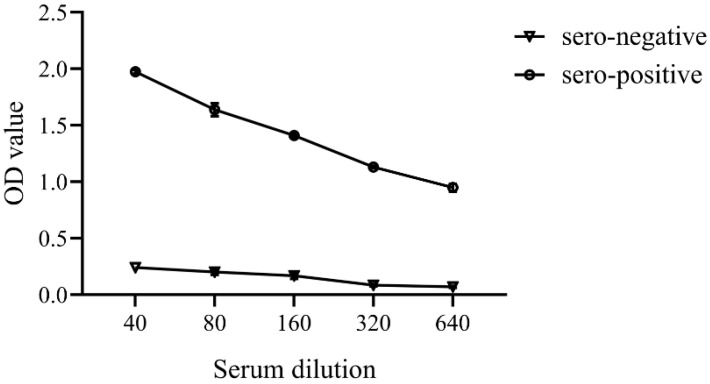
A titration curve for *Flavobacterium columnare* seropositive and seronegative serum samples (mean ± SEM) from tilapia appeared linear across the serum dilution range used in the indirect ELISA.

The optimal conditions for the ELISA were as follows: the sonicated extract antigen was used at 2.5 μg/mL in coating buffer, incubating with the antigen overnight, 1:160 diluted tilapia serum in blocking buffer (1% BSA in PBS) for incubation overnight, 1:4000 diluted of the mouse anti-tilapia IgM monoclonal antibody for 1 h and 1:2000 diluted of the anti-mouse IgG- HRP conjugated for 1 h. Average optical density values at 450 nm of the negative sera were 0.167 and the standard deviation was 0.027. From this, the cut-off point of the indirect ELISA was calculated with the mean of the negative control plus three standard deviations [17] and the value was set to 0.2480. For the interpretation, tilapia sera with an OD value higher than the cutoff value were determined as *F. columnare* antibody positive. Tilapia sera with an OD value lower than the cutoff value were considered as *F. columnare* antibody negative. A titration curve for serum appeared linear across the dilution range used for the study (Figure 3). The mean OD in the adsorbed seronegative pool (n = 15 fish, Supplementary Table S2) was 0.167 ± 0.027 and the seropositive was 1.410 ± 0.018 at the 1:160 dilution.

### 2.6. Gene Expression Determined by RT-qPCR

Spleens and head kidney were also collected at 1, 3, 14, and 21 dpv from 6 fish per group for gene expression by real time quantitative reverse transcription-polymerase chain reaction (RT-qPCR). Fish were euthanized with an overdose of clove oil anesthetic before tissue sampling for RT-qPCR. Tissues were placed immediately in RNAlater (Sigma-Aldrich, Darmstadt, Germany) and stored at 4 °C overnight. RNA-later was removed and tissues were stored at −80 °C until RNA was extracted. For this, 30 to 40 mg of gill tissue sample was used, from which RNA was extracted using a RNeasy Minikit (QIAGEN, Hilden, Germany) following the manufacturer’s instructions. RNA samples were stored at −80 °C until analyzed. RNA quantity and quality were determined using the Nanodrop ND-1000 Spectrophotometer (Thermo Fisher Scientific, Waltham, MA, USA) and adjusted to a final concentration of 1 μg μL^−1^. The mRNA was converted to complementary DNA (cDNA) using a Quantinova Reverse Transcription kit (QIAGEN) according to the manufacture’s protocol. The cDNA was analyzed for the expression of immune related genes (Table 1), including interleukin-1 (*IL-1*), tumor necrosis factor alpha (*TNFα*), MHC class 1 (*MHCI*), immunoglobulin M (*IgM*), and immunoglobulin T (*IgT*). In this study, we proposed that a biomimetic mucoadhesive nanoplatform could facilitate the bacterial antigen to potentiate the mucosal and systemic immune responses at the very beginning of the induction of the immune response. The interaction between innate and adaptive immunity was the topic of interest. The immune related genes associated with innate cytokines (*IL1β*, *TNF-α* and *MHC I*) were included. Another point regarding the MHCI gene, is that the size of the nanovaccine was as small as the intracellular organisms and lipid composition in nanoparticles can induce T cell response, as suggested by many researchers. Activation of intracellular signaling of MHC I by the nanovaccine was also included. IgT and IgM genes expression were selected to determine the systemic immune response, along with the IgM antibody titer response. We lack specific gene and antibody markers for differentiating T and B cell types in tilapia.

The RT-qPCR was performed in 96-well plates using Luna^®^ Universal qPCR master mix (New England Biolab Inc., Ipswich, MA, USA) according to the manufacturer’s instructions. Individual 20 µL reactions consisted of 10 µL Luna^®^ Universal qPCR master mix and cDNA diluted 1:10 as the template. The optimal annealing temperature for all primers was determined using the thermal gradient feature of the CFX96 Real-time PCR detection system (Bio-Rad Laboratories Inc., Hercules, CA, USA). The cycling profile was as follows: enzyme activation was carried out at 95 °C for 1 min, followed by 45 cycles of denaturing at 95 °C for 15 s, and annealing and primer extension at 55 and 60 °C for 30 s. β-actin was used as an internal control for cDNA normalization. Gene expression was calculated relative to the β-actin using the 2−∆∆Ct method [19,20]. The gene expression data were normalized to the reference genes β-actin and expressed as a comparison of vaccinated fish relative to control fish [18,21,22,23]. The amplification efficiency of all primer pairs was assessed before performing the RT-qPCR analysis with an average amplification efficiency of 90.01–114.87 % using the equation: E = −1 + 10^(−1/slope)^.

### 2.7. Statistical Analysis

All analyses were performed with GraphPad Prism Software (Version 8.0), Inc. (San Diego, CA, USA). Normality was checked using the Shapiro-Wilk test. The mean ± SE and analyzed with ANOVA followed by post-hoc Bonferroni’s test for multiple comparisons: among groups at each time point and among trial periods within each group. A value of *p* < 0.05 was regarded as statistically significant. 

## 3. Results

### 3.1. Vaccine Efficacy

**Figure 4:**
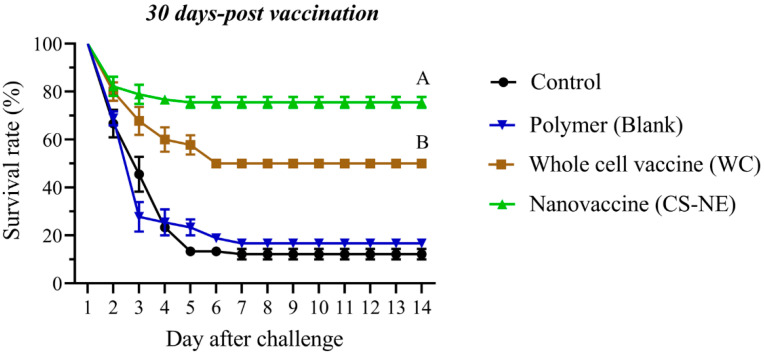
The percentage survival in vaccinated fish after immersion challenge with 1 × 10^7^ CFU/mL *F. columnare* (strain F-K17/1), i.e., non-vaccinated, polymer, WC and CS-NE groups (30 from each group, 3 replicate tanks). The CS-NE vaccinated fish showed significantly higher levels of survival. Different capital letters indicate significant differences (*p* < 0.05) among groups at 14 days post- challenge. One-way analysis of variance, and repeated measures analysis of variance followed by Bonferroni’s multiple comparison test were used for statistical analysis.

No fish died in any of the groups during the 30 days post-immersion vaccination. After challenging fish with *F. columnare* by immersion at 30 days post vaccination (dpv), the onset of mortality was observed during the early phase (1–5 days) of infection in all vaccinated and non-vaccinated fish. The moribund fish exhibited a clinical sign of skin color change, lesion on the body, hemorrhages, and/or deteriorated tail. In order to assess the vaccine’s efficacy, an RPS value greater than 60% was considered to be a protective effect from the vaccination [13,24]. The percentage survival of vaccinated and control fish after challenge is shown in Figure 4; the RPS value of CS-NE group was the highest and greater than 72. However, a group vaccinated with whole-cell bacteria had an RPS value of 43. The mortality in the non-vaccinated fish was 87% (RPS = 0).

### 3.2. Serum Bactericidal Activity (SBA)

**Figure 5:**
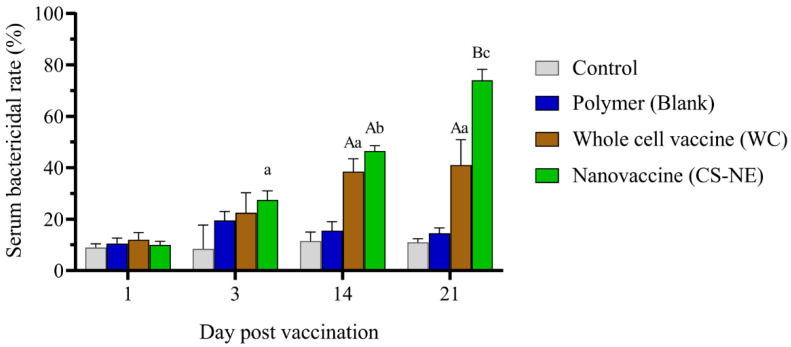
Serum bactericidal activity (SBA) of vaccinated fish. SBA of sera from CS-NE vaccinated were significantly higher than control groups at 1, 3, 14, and 21 days post-vaccination. Different capital letters indicate significant differences between groups at *p* < 0.05 within each time point. Different small letters indicate significant differences over time within each group (N = 6). Two-way analysis of variance, and repeated measures analysis of variance followed by Bonferroni’s multiple comparison test were used for statistical analysis.

Serum bactericidal activity was the lowest in PBS and the highest in CS-NE group. The SBA of sera from CS-NE vaccinated fish was significantly elevated above that of the WC and control groups at 1, 3, 14, and 21 dpv (*p <* 0.05) (Figure 5, Supplementary Table S3). The number of bacterial colonies obtained after treatment with sera from the CS-NE and WC groups was significantly lower than the control groups.

### 3.3. Enzyme-Linked Immunosorbent Assay (ELISA) 

**Figure 6:**
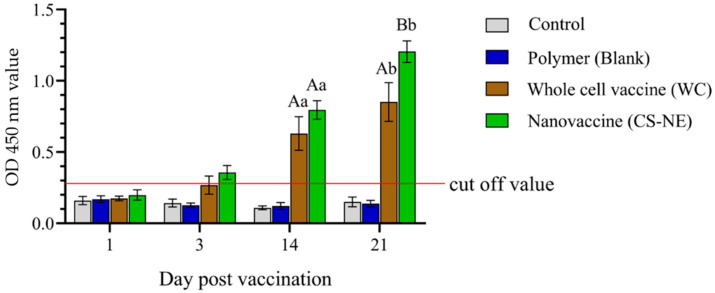
Identification of seropositive and seronegative fish amongst vaccine groups using an indirect ELISA. Different capital letters indicate significant differences at *p* < 0.05 among groups at each time point. Different small letters indicate significant differences over time within each group (N = 6). Two-way analysis of variance, and repeated measures analysis of variance followed by Bonferroni’s multiple comparison test were used for statistical analysis.

Serum samples were collected from each group to assess antibody levels at 1, 3, 14, and 21 days post-vaccination (6 fish/group/time), respectively. We found that tilapia sera were positive at 3, 14, and 21 dpv in the WC and CE-NE groups, with sera from the CS-NE group having the highest OD value between all groups at 21 dpv. Specific IgM levels were significantly greater in vaccinated fish compared to control fish (*p* < 0.001) at 14 and 21 dpv (Figure 6, Supplementary Table S4), and which was seen to increase between 14 and 21 dpv. 

### 3.4. Gene Expression with RT-qPCR 

**Figure 7:**
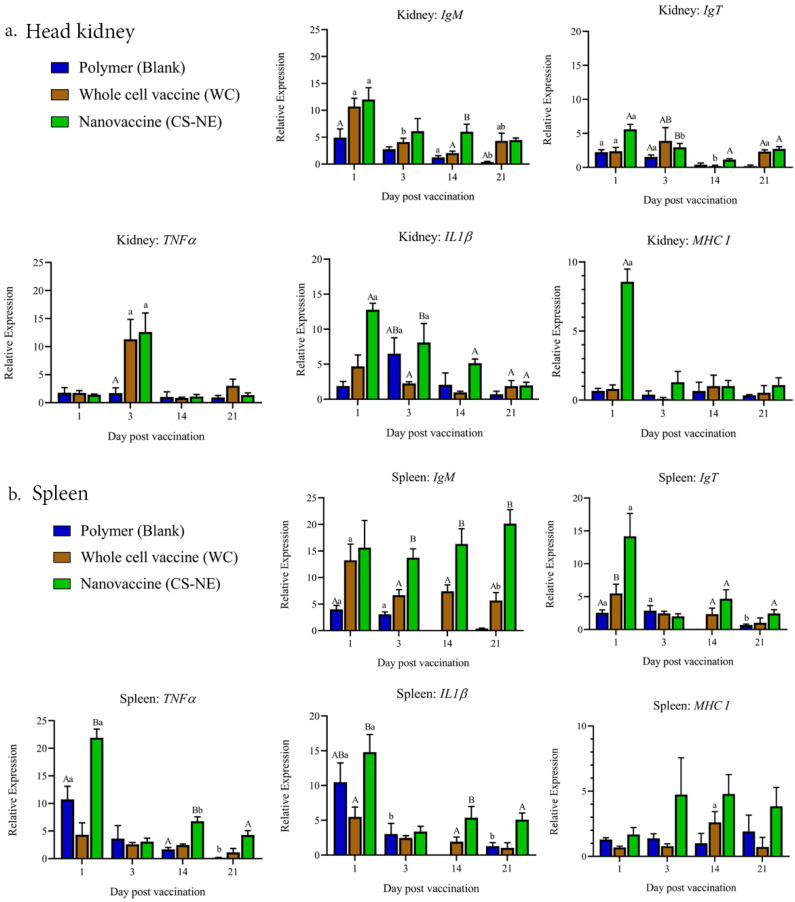
Gene expression of five immune genes—*IgM*, *IgT*, *IL1β*, *TNF-α*, and *MHCI* in the head kidney (**a**) and spleen (**b**) of vaccinated fish (CS-NE (green), WC (brown), Polymer (blue)) relative to unvaccinated control fish at 1, 3, 14, and 21 days post-vaccination. Different capital letters indicate significant differences at *p* < 0.05 among groups at each time point. Different small letters indicate significant differences over time within each group (N = 6). Two-way analysis of variance, and repeated measures analysis of variance followed by Bonferroni’s multiple comparison test were used for statistical analysis.

At 1, 3, 14, and 21 dpv, the expression of 5 immune-related genes i.e., *IgM*, *IgT*, *IL1β*, *TNF-α*, and *MHCI*, was examined in the spleen and head kidney of fish from each experimental group. Expression of *IgM*, *IgT*, *IL1β*, *TNF-α*, and *MHCI* was significantly higher in the CS-NE group compared to the WC and polymer vaccinated groups (Figure 7, Supplementary Table S5). Genes encoding *IgM*, *IgT, IL1β*, *TNF-α*, and *MHCI* genes were also upregulated at each time point examined, especially in the CS-NE vaccinated group. The expression of *MHCI*, *TNFα*, and *IL-1* genes was highly upregulated at the early stage of vaccination induction (1 and 3 dpv) and gradually declined in both head kidney and spleen of vaccinated fish. This expression kinetics was clearly seen in the CS-NE vaccinated fish when compared to the control fish. The *MHCI* gene expression tended to be upregulated until 21 dpv in the spleen of CS-NE vaccinated fish. The expression of IgT and IgM was also higher at the initial stages of vaccination in the head kidney of vaccinated fish in all groups and was reduced in the later stages of the trial (21 dpv). However, the expression of IgM was consistently upregulated in the spleen of CS-NE vaccinated fish throughout the 21-day experimental period. 

## 4. Discussion

An understanding of the adaptive immune response following vaccination is needed to develop a safe and efficacious vaccine against columnaris disease in tilapia, including knowledge on the specific antibody kinetics to the vaccine. Our previous study showed that a biomimetic mucoadhesive nanovaccine was able to activate the mucosal associated lymphoid tissues in vaccinated fish and generated a strong mucosal immune response against columnaris disease in the fish [4]. In this study, we investigated the specific anti-*F. columnare* IgM response in tilapia immunized with the vaccine, together with their ability to resist experimental challenge with a virulent strain of *F. columnare*. Bactericidal serum activity and the use of an ELISA developed in-house were used as serological assays for evaluation of the systemic immune response in fish vaccinated with our mucosal delivery-nanovaccine. This vaccine performed better than the WC immersion vaccine, reflected by the RPS values obtained after performing a *F. columnare* immersion challenge, with the biomimetic-mucoadhesive nanovaccine providing significantly better protection against the pathogen. This finding confirms the results of our previous study and the average percentage survival obtained with this vaccine in the two trials was 72–78% for immersion vaccinated tilapia held at 25–28 °C and challenged with 1.0 × 10^7^ *F. columnare* CFU per fish, regardless of their age and size (average size of the fish used in previous study was 5 g compared with 100 g fish used in the present study).

The ELISA is recognized as a sensitive, widely used assay, reliable for the detection and quantification of specific humoral antibody responses to fish pathogens [25,26,27] including *F. columnare* [8,11]. In this study, the ELISA protocol was optimized and validated using positive and negative control serum. The positive serum samples were collected from tilapia experimentally infected with the pathogen, whereas the negative control sera were collected from the same fish prior to the challenge as well as non-challenge fish. Although very low OD values were observed in the negative control fish, this might be explained by non-specific binding associated with tilapia serum [8,11,28]. In fact, fish used in this study were not specific pathogen-free, and the possibility of pre-exposure to *F. columnare* could not be ruled out. Multiple factors can influence the dynamic patterns of the specific antibody responses obtained, such as the pathogen, type of vaccines used, the delivery method, and the inclusion of an adjuvant or delivery vehicles in the vaccine. For example, in one study, it was noted that the antibody response of tilapia vaccinated with a formalin killed the *F. columnare* vaccine containing Freund’s complete adjuvant occurred within 14 dpv and then gradually declined by 21 and 28 dpv [11], while little or no antibody response was seen in Nile tilapia fingerlings receiving a *F. columnare* vaccine delivered by oral or immersion vaccination, again using formalin-killed bacteria [12]. Contrary to this, in rainbow trout, immersion vaccination is able to induce a high serum IgM antibody response up to 21 to 28 dpv, after which it is gradually reduced. A greater IgM response was detected in the skin mucus and persisted up to 4 to 6 weeks post-immersion vaccination with a live attenuated *F. columnare* vaccine, as well as a recombinant *F. columnare* DnaK protein vaccine [29]. In our study, we detected a rapid antibody response as early as 3–7 dpv, which were significantly higher at 14 to 21 dpv in the CS-NE vaccinated tilapia. Peak antibody levels were reached by 21 days post-immersion vaccination. The study showed that vaccinated tilapia are capable of mounting a significant humoral immune response to *F. columnare*, but this was only seen when the bacteria were incorporated into the charged-mucoadhesive nano-delivery system.

Serum bactericidal activity has been used as a measure of complement-mediated activity in the presence of vaccine-induced antibodies. Although the evaluation of serum antibacterial activity is considered to be a non-specific response for inhibiting the growth of bacteria [30,31], it is accepted as an in vitro correlate of protection for the evaluation of the immunogenicity of bacterial vaccines [30,32,33]. The increased serum bactericidal activity detected after immersion vaccination reflects the raise of protective proteins in the serum, including immunoglobulins, proteins of the complement system, acute phase proteins, cytokines, lysozyme, transferrin, and lectins that are usually induced or elevated after infection or vaccination [31,34]. Humoral immune responses by ELISA-specific IgM antibodies were significantly higher in CS-NE vaccinated fish, in accordance with a significantly higher SBA, indicating stimulation of the immune response against *F. columnare* by the mucoadhesive nanovaccine. A rapid and robust method for assessing serum bactericidal activity may inform the best vaccine formulation, optimal dose, and schedule for a vaccine, and may be a useful part of evaluating large vaccine efficacy trials [35]. In our previous study [4], we showed evidence for a systemic cellular immune response, indicated by the hyperplasia of lymphoid cells in spleen and head kidney in the nanovaccine immunized group. Researchers demonstrated that lipid nanoparticles could induce T cell responses, regulating adaptive cell-mediated immunity, resulting in protection against intracellular pathogens [36,37,38,39]. However, due to the lack of molecular markers and antibodies to specifically identify T and B cells in tilapia, it is difficult to investigate the relationship between specific cellular immune responses elicited by the CS-NE vaccine and their role in protective systemic immune responses.

The nanovaccine modulated the expression of *TNF-α* or *IL1β*, two key pro-inflammatory cytokines that are crucial for coordinating cell-mediated immunity and play a critical role in modulating the immune system against *F. columnare* infection [40,41]. Our previous studies confirmed that macrophages, intra-epithelial lymphocytes, and eosinophilic granular cells are recruited to the sites of infection, with the aim of controlling and eradicating the *F. columnare* infection, as seen in MALT histopathology during the early stages of response to the infection (3 dpi) [4]. There is a complex interaction between innate and adaptive immune responses. The set of immune-related genes selected in the present study potentially reflect the complexity of this relationship. Upregulation of *MHCI, TNFα* and *IL-1* are mediators of the innate immune response, orchestrating the innate cell to respond to the infection, and activating the protective adaptive immune response. In this study, MHC-I gene expression was highly upregulated in the head kidney and spleen of the CS-NE vaccinated fish, suggesting the potential intracellular antigen presented by polymeric nanoparticles as mentioned by researchers [42,43]. Lipid nanoparticles are able to induce T cell responses and regulate adaptive cell-mediated immunity, resulting in protection against intracellular pathogens [36,37,38,39].

During initiation of adaptive immune mechanisms, early immune related gene expression responses are necessary, as they provide the first line of defense against the infection or vaccination [44], and we focused our study on 1 to 21 dpv, when development of the immune response was ongoing. The expression profile of immune related genes in lymphoid organs of vaccinated fish demonstrate the immunological cascade of antibody production by both IgM and IgT-producing B cells in response to the *F. columnare* immersion vaccination. Similar higher levels of immune gene expression were seen in spleen and head kidney of CS-NE vaccinated tilapia throughout the post-vaccination periods when compared with the other groups. A striking result of our study is the implication of B cells expressing *IgT* and *IgM* genes in the spleen in response to the immersion vaccination at 24 h post-vaccination and 24 h to 21 dpv, respectively. The results support the available information that immersion vaccination induces splenic and kidney IgT responses in tilapia. The question of the role of *IgT* gene expression in the spleen of *F. columnare*-immersion vaccinated fish is intriguing. The dominant expression of the *IgM* and *IgT* transcripts in vaccinated fish and the increase of serum IgM concentration upon vaccination might indicate that the serum IgM and/or IgT antibodies were likely produced by splenic IgM or IgT producing cells, respectively. Alternatively, it might be speculated that specific IgT producing B cells may become activated and proliferate before migrating to the mucosal sites and may be found in the spleen as transiting cells [45]. IgT and IgM played an important role against *F. columnare* infection in gill mucosal and the systemic immunity of rainbow trout [44]. Due to a lack of available monoclonal antibodies against the IgT immunoglobulin class in tilapia, similar studies are limited in tilapia. Ideally, IgT-ELISA studies should be used to investigate the persistence of the immune response in fish vaccinated with the mucoadhesive nano-vaccine. We showed that CS-NE can induce tilapia IgT, and IgM responses in vaccinated fish and promote intracellular uptake by MHC-I, and regulate adaptive cell-mediated immunity to provide protection against *F. columnare* infection. However, the duration of protection and the anamnestic response of the IgT and IgM antibodies should be investigated after challenging or a booster vaccination over the lifespan of vaccinated tilapia. The reactivation of memory B cells in lymphoid organs by the chitosan-lipid based mucoadhesive nano-platforms should also be examined.

## 5. Conclusions

We applied an innovative nanotechnology to develop a mucosal vaccine delivery system suitable for improved immersion vaccination of tilapia. The biomimetic nanoparticles induced a strong humoral immune response, resulting in a significant increase in RPS against columnaris disease. The splenic IgM and IgT genes were highly upregulated, corresponding with higher serum IgM production and a greater serum bactericidal activity against the homologous challenge strain. The results confirm that the charged-mucoadhesive nanovaccine modified by a chitosan-based delivery is an effective alternative platform for immersion vaccination of tilapia, generating systemic immune responses against columnaris disease in tilapia.

## Figures and Tables

**Table 1:** Primers used in RT-qPCR.

Gene	Target	Sequence Forward/Reverse (5′-3′)	Product (bp)	Reference
β-actin F β-actin R	Housekeeping gene	AAGGACCTGTACGCCAACAC ACATCTGCTGGAAGGTGGAC	196	[18]
TNFα F TNFα R	Inflammation related gene	CTCACAGATAGCGGCATCAA CCTGGGCTCTCTCTGTGTTC	190	[18]
MHC Iiβ F MHC Iiβ R	Adaptive immune-related gene	TCAGCACAGCAGATGGATTC GCCTGCTTCACTCCAAACTC	175	[18]
IL-1β F IL-1 β R	Adaptive immune-related gene	AAGATGAATTGTGGAGCTGTGTT AAAAGCATCGACAGTATGTGAAAT	175	[4]
IgM-F IgM-R	Adaptive immune-related gene	TGGTACTGGGGGTCAAACAT TAAGCGATCCATTCCAGTCC	156	[18]
IgT-F IgT-R	Adaptive immune-related gene	AGACACACCAGAGTGATTTCAT AGACACACCAGAGTGATTTCATCAG	78	[4]

## Supplementary Materials

The following are available online at https://www.mdpi.com/article/10.3390/vaccines9111253/s1, Table S1: Standardization of the indirect ELISA, Table S2: A titration data (OD value at 450 nm) for seronegative serum and seropositive, Table S3: The number of viable bacteria, Table S4: OD 450 nm value of serum samples from each group, Table S5: the expression of 5 immune-related genes was examined in the spleen and head kidney.
