# Supplementary material for: Immersion Vaccination by a Biomimetic-Mucoadhesive Nanovaccine Induces Humoral Immune Response of Red Tilapia (Oreochromis sp.) against Flavobacterium columnare Challenge
Source: Vaccines (Basel). 2021 Oct 29;9(11):1253. doi: 10.3390/vaccines9111253 (PMC8624005; doi:10.3390/vaccines9111253)
Supplement: Supplementary file 1 [file vaccines-09-01253-s001.zip › vaccines-1395991-supplementary.pdf]

Supplementary Table S1: Standardization data of the indirect ELISA

Antigen 2 fold dilution (ug/ml)		20	10	5	2.5**	1.25	PBS	20	10	5	2.5**	1.25	PBS
Serum 2 fold dilution	1:20	0.8642	0.4235	0.4103	0.4012	0.3897	0.2344	2.3793	2.3421	2.1095	1.9867	1.6782	0.6811
	1:40	0.7314	0.3541	0.3243	0.3045	0.2314	0.2152	2.2134	2.1038	1.9873	1.8643	1.4978	0.5371
	1:80	0.4712	0.3157	0.2376	0.2287	0.2134	0.2008	2.1342	1.8723	1.5972	1.6722	1.3421	0.2972
**	1:160	0.2971	0.1987	0.1863	0.1673	0.1583	0.1132	1.9782	1.5347	1.4222	1.3147	1.2457	0.1253
	1:320	0.1978	0.1871	0.1781	0.1559	0.1357	0.1103	1.8631	1.2473	1.1243	0.8972	0.5782	0.1324
	1:640	0.1876	0.1688	0.1534	0.1422	0.1401	0.1211	1.3425	0.6978	0.9718	0.6472	0.3223	0.1291
	1:1280	0.1301	0.1253	0.1182	0.1073	0.1033	0.1019	0.7984	0.6472	0.6312	0.5124	0.2147	0.1732
	PBS	0.1075	0.1102	0.1031	0.0981	0.1092	0.0987	0.3241	0.2479	0.1247	0.1282	0.1103	0.1007
		negative serum						positive serum					

Antigen 2 fold dilution (ug/ml)		20	10	5	2.5**	1.25	PBS	20	10	5	2.5**	1.25	PBS
Serum 2 fold dilution	1:20	0.9123	0.4813	0.4031	0.3972	0.3811	0.2293	2.3793	2.3421	2.1095	1.9867	1.6782	0.6811
	1:40	0.8212	0.3782	0.3456	0.2789	0.2245	0.2271	2.2134	2.1038	1.9873	1.8643	1.4978	0.5371
	1:80	0.5213	0.2913	0.2731	0.2109	0.1973	0.2017	2.1342	1.8723	1.5972	1.6722	1.3421	0.2972
**	1:160	0.311	0.2013	0.1934	0.1732	0.1583	0.1132	1.9782	1.5347	1.4222	1.3147	1.2457	0.1253
	1:320	0.2013	0.1834	0.1697	0.1521	0.1322	0.1239	1.8631	1.2473	1.1243	0.8972	0.5782	0.1324
	1:640	0.1791	0.1639	0.1522	0.1397	0.1287	0.1173	1.3425	0.6978	0.9718	0.6472	0.3223	0.1291
	1:1280	0.1301	0.1253	0.1182	0.1073	0.1033	0.9917	0.7984	0.6472	0.6312	0.5124	0.2147	0.1732
	PBS	0.0978	0.1017	0.0983	0.1005	0.1038	0.092	0.3241	0.2479	0.1247	0.1782	0.1678	0.1572
		negative serum						positive serum					

Supplementary Table S2: Data of the indirect ELISA-OD value at 450 nm of *F. columnare*-seronegative and -seropositive tilapia sera.

Serum	Replicate 1					Replicate 2				
	1:40	1:80	1:160	1:320	1:640	1:40	1:80	1:160	1:320	1:640
Seronegative 1	0.2209	0.193	0.1694	0.0799	0.0648	0.2236	0.1931	0.1511	0.0813	0.0671
Seronegative 2	0.2788	0.2432	0.2333	0.0712	0.0614	0.2904	0.2371	0.2065	0.077	0.0671
Seronegative 3	0.1857	0.1685	0.1365	0.0747	0.0606	0.2059	0.1642	0.1266	0.0765	0.0738
Seronegative 4	0.2676	0.2163	0.1721	0.0818	0.0564	0.2649	0.2194	0.1847	0.0944	0.0707
Seronegative 5	0.2669	0.2197	0.1709	0.0695	0.0613	0.2556	0.2112	0.1717	0.0812	0.063
Seronegative 6	0.2662	0.2362	0.1762	0.0677	0.0532	0.282	0.2489	0.2082	0.0795	0.0675
Seronegative 7	0.2113	0.1991	0.1772	0.0817	0.0707	0.1941	0.1888	0.1609	0.0723	0.0633
Seronegative 8	0.2267	0.1864	0.1508	0.1141	0.0964	0.2317	0.1966	0.1336	0.0963	0.0892
Seronegative 9	0.2632	0.2127	0.1777	0.0785	0.0565	0.2884	0.2024	0.1730	0.091	0.0741
Seronegative 10	0.2549	0.2127	0.1795	0.083	0.0755	0.2469	0.1819	0.1491	0.0857	0.0795
Seronegative 11	0.2158	0.1873	0.1524	0.0885	0.0776	0.2027	0.1697	0.1244	0.0792	0.0688
Seronegative 12	0.2242	0.1708	0.1437	0.0807	0.0651	0.2155	0.1915	0.1659	0.091	0.0801
Seronegative 13	0.2292	0.1827	0.1621	0.0892	0.078	0.2323	0.195	0.1530	0.0878	0.0623
Seronegative 14	0.2186	0.1802	0.1598	0.0899	0.0745	0.2026	0.1526	0.1241	0.0756	0.0616
Seronegative 15	0.274	0.2486	0.2131	0.11	0.0732	0.2606	0.2305	0.2025	0.1034	0.0725
Seropositive 1	1.9873	1.5972	1.4222	1.1243	0.9718	1.9612	1.678	1.397	1.1378	0.924

Mean (Seronegative)	SD	3SD
0.1670	0.0270	0.0810

Supplementary Table S3: Data of the number of viable bacteria (CFU/mL) and bactericidal rate. Bactericidal rate was calculated as follows: (1 − the number of viable bacteria after serum treatment/the number of viable bacteria after PBS treatment) × 100% [16].

Day		PBS control		Group							
		PBS		control		polymer		WC		CS-NE	
1	CFU/mL	9.8x10*7	1.03x10*8	9.0x10*7	9.3x10*7	8.6x10*7	9.4x10*7	8.4x10*7	9.3x10*7	8.9x10*7	9.2x10*7
	Bactericidal rate	-	-	8	10	12	9	14	10	9	11
3	CFU/mL	1.05x10*8	9.3x10*7	8.9x10*7	9.1x10*7	8.7x10*7	7.3x10*7	7.6x10*7	7.7x10*7	7.4x10*7	7.0x10*7
	Bactericidal rate	-	-	15	2	17	22	28	17	30	25
14	CFU/mL	1.13x10*8	1.02x10*8	9.7x10*7	9.3x10*7	9.8x10*7	8.4x10*7	6.5x10*7	6.6x10*7	5.9x10*7	5.6x10*7
	Bactericidal rate	-	-	14	9	13	18	42	35	48	45
21	CFU/mL	9.7x10*8	1.12x10*7	8.7x10*7	9.9x10*7	8.1x10*7	9.7x10*7	6.4x10*7	5.8x10*7	2.8x10*7	2.6x10*7
	Bactericidal rate	-	-	10	12	16	13	34	48	71	77

Supplement Table S4: Data of serum OD value (450 nm) from tilapias in each group at different time points after vaccination.

Day 1	Control	WC	CS-NE	Polymer
	0.1549	0.1820	0.1990	0.1870
	0.1699	0.1706	0.2439	0.1434
	0.1743	0.1893	0.1654	0.1927
	0.1387	0.1882	0.1943	0.1779
	0.1192	0.1441	0.2336	0.1388
	0.2001	0.1717	0.1556	0.1784
Day 3	Control	WC	CS-NE	Polymer
	0.1437	0.1959	0.3858	0.1337
	0.1121	0.3005	0.3293	0.1021
	0.1629	0.3252	0.3015	0.1349
	0.1146	0.1768	0.3601	0.1145
	0.1355	0.3026	0.4368	0.1306
	0.1835	0.3073	0.3265	0.1451
Day 14	Control	WC	CS-NE	Polymer
	0.1154	0.6252	0.7728	0.1225
	0.1162	0.6971	0.8548	0.1081
	0.1117	0.4872	0.7174	0.1544
	0.1040	0.7030	0.8026	0.1190
	0.0825	0.7734	0.8840	0.0845
	0.1192	0.4940	0.7425	0.1406
Day 21	Control	WC	CS-NE	Polymer
	0.1929	0.7312	1.2051	0.1521
	0.1549	1.0284	1.1005	0.1550
	0.1237	0.7744	1.2691	0.1314
	0.1861	0.7626	1.2345	0.1644
	0.1395	1.0197	1.1314	0.1205
	0.1059	0.7921	1.2897	0.1030

Supplement Table S5: Data of gene expression of 5 immune genes - IgM, IgT, IL1β, TNF-α and MHCI in the head kidney (a) and spleen (b) of vaccinated fish [CS-NE, WC, Polymer relative to unvac-cinated control fish at 1, 3, 14, 21 days post-vaccination

Head-kidney

IgM	Time	Mean Fold Change in gene expression	SE
WC	Day 1	10.70	1.55
	Day 3	4.09	0.73
	Day 14	2.04	0.39
	Day 21	4.33	1.43
CS-NE	Day 1	12.02	2.20
	Day 3	6.12	2.35
	Day 14	6.01	1.43
	Day 21	4.47	0.40
Polymer	Day 1	4.93	1.62
	Day 3	2.79	0.46
	Day 14	1.24	0.33
	Day 21	0.40	0.12

IgT	Time	Mean Fold Change in gene expression	SE
WC	Day 1	2.37	0.57
	Day 3	3.89	1.97
	Day 14	0.18	0.14
	Day 21	2.33	0.27
CS-NE	Day 1	5.60	0.74
	Day 3	2.96	0.55
	Day 14	1.39	0.43
	Day 21	2.73	0.33
Polymer	Day 1	2.24	0.37
	Day 3	1.55	0.30
	Day 14	0.39	0.24
	Day 21	0.19	0.18

TNFα	Time	Mean Fold Change in gene expression	SE
WC	Day 1	1.74	0.43
	Day 3	11.30	3.57
	Day 14	0.85	0.14
	Day 21	3.01	1.18
CS-NE	Day 1	1.43	0.12
	Day 3	12.59	3.42
	Day 14	1.10	0.37
	Day 21	1.37	0.39
Polymer	Day 1	1.78	0.92
	Day 3	1.71	0.93
	Day 14	1.04	0.90
	Day 21	0.92	0.37

IL1β	Time	Mean Fold Change in gene expression	SE
WC	Day 1	4.69	1.63
	Day 3	2.26	0.23
	Day 14	0.97	0.18
	Day 21	1.88	0.80
CS-NE	Day 1	12.78	0.93
	Day 3	8.10	2.72
	Day 14	5.13	0.63
	Day 21	1.97	0.45
Polymer	Day 1	1.89	0.66
	Day 3	6.50	2.27
	Day 14	2.08	1.67
	Day 21	0.70	0.43

MHCI	Time	Mean Fold Change in gene expression	SE
WC	Day 1	0.81	0.29
	Day 3	0.09	0.11
	Day 14	1.02	0.79
	Day 21	0.54	0.53
CS-NE	Day 1	8.56	0.93
	Day 3	1.30	0.78
	Day 14	1.02	0.41
	Day 21	1.09	0.53
Polymer	Day 1	0.67	0.19
	Day 3	0.39	0.28
	Day 14	0.66	0.63
	Day 21	0.35	0.05

Spleen

IgM	Time	Mean Fold Change in gene expression	SE
WC	Day 1	13.24	3.06
	Day 3	6.67	1.08
	Day 14	7.40	1.24
	Day 21	5.67	1.51
CS-NE	Day 1	15.61	5.15
	Day 3	13.72	1.68
	Day 14	16.33	2.87
	Day 21	20.14	2.65
Polymer	Day 1	4.00	0.76
	Day 3	3.09	0.47
	Day 14	0.01	0.01
	Day 21	0.39	0.12

IgT	Time	Mean Fold Change in gene expression	SE
WC	Day 1	5.50	1.40
	Day 3	2.47	0.33
	Day 14	2.35	0.91
	Day 21	1.03	0.75
CS-NE	Day 1	14.17	3.49
	Day 3	1.99	0.43
	Day 14	4.67	1.39
	Day 21	2.45	0.57
Polymer	Day 1	2.55	0.44
	Day 3	2.88	0.76
	Day 14	0.02	0.01
	Day 21	0.71	0.17

TNFα	Time	Mean Fold Change in gene expression	SE
WC	Day 1	4.31	2.18
	Day 3	2.59	0.34
	Day 14	2.45	0.20
	Day 21	1.13	0.71
CS-NE	Day 1	21.91	1.58
	Day 3	6.16	1.28
	Day 14	6.78	0.80
	Day 21	4.29	0.81
Polymer	Day 1	10.73	2.39
	Day 3	3.63	2.35
	Day 14	1.68	0.36
	Day 21	0.19	0.04

IL1β	Time	Mean Fold Change in gene expression	SE
WC	Day 1	5.50	1.40
	Day 3	2.47	0.33
	Day 14	1.93	0.66
	Day 21	1.03	0.75
CS-NE	Day 1	14.80	2.55
	Day 3	5.88	0.39
	Day 14	5.38	1.60
	Day 21	5.10	0.96
Polymer	Day 1	10.48	2.77
	Day 3	3.99	1.10
	Day 14	0.01	0.00
	Day 21	1.28	0.50

MHCI	Time	Mean Fold Change in gene expression	SE
WC	Day 1	0.69	0.10
	Day 3	0.78	0.19
	Day 14	2.61	0.82
	Day 21	0.72	0.74
CS-NE	Day 1	1.68	0.53
	Day 3	4.74	2.82
	Day 14	4.78	1.50
	Day 21	3.85	1.45
Polymer	Day 1	1.29	0.15
	Day 3	1.38	0.36
	Day 14	1.00	0.77
	Day 21	1.91	1.25
